# A higher-output grass-based spring-calving dairy production system in Ireland—Factors influencing performance variability over a 9-year period

**DOI:** 10.3168/jdsc.2025-0967

**Published:** 2026-03-27

**Authors:** K.M. Pierce, Z.C. McKay, K.M. McCarthy, A.G. Fahey, M. Wallace, M.B. Lynch, N.A. Walsh, N. Ryan, N. Dooley, F.J. Mulligan

**Affiliations:** 1 School of Agriculture and Food Science, University College Dublin, Belfield, Dublin 4, Ireland; 2 School of Veterinary Medicine, University College Dublin, Belfield, Dublin 4, Ireland; 3 Teagasc Environment Research Centre, Johnstown Castle, Wexford, Ireland, Y35 Y521; 4 University College Dublin Lyons Research Farm, Lyons Estate, Celbridge, Naas, Co. Kildare, Ireland W23 ENY2; 5 Golden Vale Research, Clonmel, Co. Tipperary, Ireland, E91YY38

## Abstract

The competitiveness of the Irish dairy industry relies on the efficient utilization of grazed grass. Growth in milk output over the last decade was primarily achieved by increasing herd size rather than cow yield. However, limitations on land, labor, and environmental regulations restricting stocking rates mean higher concentrate input grazing systems, which focus on increased output per cow, merit consideration. From 2016, a multiyear study commenced at University College Dublin investigating a higher-output grazing system, characterized by increased milk solids (MSo; fat kg + protein kg) output and higher concentrate supplementation per cow, by integrating the most recent advances in grassland management, nutrition, and genetics. Approximately 57 spring-calving dairy cows were selected and managed as one herd. The 9-year average herbage yield was 12,560 kg DM/ha, and grass utilization (>4 cm) was 88.4%. Annual grazed grass intake per cow averaged 2,931 kg DM. Interannual variability in daily grass growth and total DM grown led to a wide range in grazed grass consumed per cow (2,260–3,410 kg DM). On average, cows were allocated 1,906 kg DM of grass silage and 1,299 kg DM of concentrate. Farm-gate nitrogen (N) use efficiency, used to estimate the ratio of N input versus output in farm produce, averaged 35% but increased in years when fertilizer use was reduced. Average MSo production (583 kg/cow) and the 6-wk in-calf rate (76.4%) both exceeded national averages over the same period. This study highlights the potential of higher-output grass-based systems to achieve efficient grassland performance and high levels of MSo production per cow. Future research should focus on strategies to improve interyear performance consistency of milk production, fertility, and grassland management, particularly in managing the impacts of climatic variation affecting grass growth. This may include more flexible and responsive concentrate supplementation approaches to offset yield depressions during periods of reduced grass availability.

## Highlights

• Average milk solids production was 583 kg/cow, greater than the national average (439 kg).
• The mean 6-week in-calf rate was 76.4 %, exceeding national averages over the same period.
• The system maintained high herbage yield (12,560 kg DM/hectare) and utilization (88.4%).
• These data highlight the sensitivity of performance to interannual climate and grass growth variability.

## Graphical Abstract

This article examines the 9-year average performance of the University College Dublin Lyons high-output grazing system. On average, the herd produced 583 kg of milk solids (fat kg + protein kg) per cow, while maintaining a 6-week in-calf rate of 76.4%. Annual herbage production averaged 12.6 tonnes of dry matter (DM) per hectare, and cows consumed 2.9 tonnes of grazed grass DM on average annually. CP = crude protein; NDF = neutral detergent fiber; OMD = organic matter digestibility; SR = stocking rate.

**Figure:**
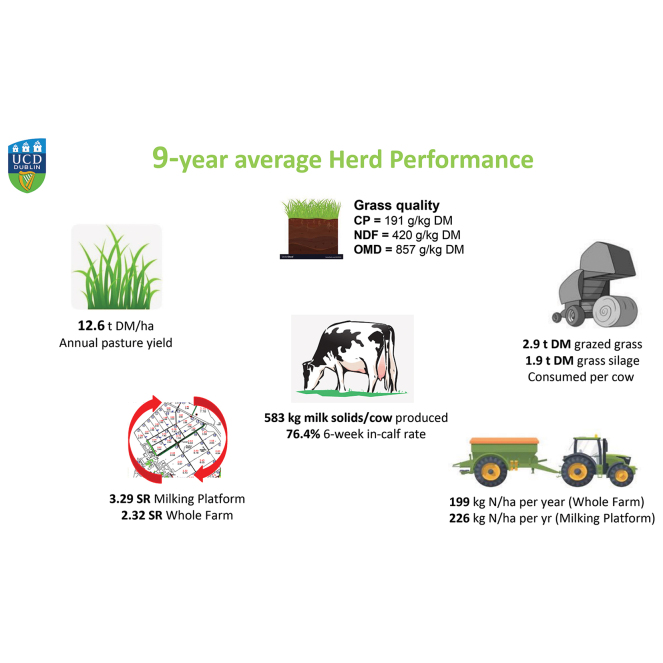

The removal of European Union (**EU**) milk quotas in 2015 enabled significant expansion in Ireland's dairy sector. Historically, grazing systems in Ireland were designed to maximize the utilization of grazed grass and were characterized by relatively low output per cow (Kelly et al., 2020). Between 2014 and 2022, national milk production increased by ~56% (Dillon et al., 2025), and dairy cow numbers grew by ~41% from 2013 to 2023 (EPA, 2024). The dairy industry now contributes an estimated €17 billion annually to the Irish economy (Ernst and Young, 2023), with exports valued at €6.3 billion (Bord Bia, 2024). However, environmental restrictions, particularly the legally binding Climate Action Plan (Government of Ireland, 2023) and the European nitrates derogation legislation (DAFM, 2025), may limit the scope for future growth in milk output through additional cow numbers. Consequently, grazing dairy production systems based on higher output per cow merit consideration, as potentially achieving national milk production with fewer dairy cows, could help reduce agricultural GHG emissions and nutrient loss associated with dairy production systems in Ireland. Such systems may also offer advantages where land, infrastructure, and skilled labor are limited. In this context, we discuss a multiyear study conducted in University College Dublin (**UCD**), investigating the performance metrics required for a sustainable higher-output grass-based spring milk production system, characterized by moderate concentrate supplementation. This report will discuss the findings over a 9-yr period (2016–2024). The objective of the study was to incorporate the most recent advances in grassland management, dairy cow nutrition, and genetics, and investigate factors affecting the performance of a higher-output grass-based spring-calving dairy herd in Ireland.

The study herd was assembled in November 2015 at the UCD Lyons Farm Celbridge, Naas, Co. Kildare, Ireland (53°29′N, 6°53′W, 80 m above sea level). Initially, 60 multiparous spring-calving Holstein Friesian dairy cows were sourced from Irish dairy farms and from the existing dairy herd at UCD Lyons Farm. The breeding objective of this study was to select a genotype that could enable high milk solids (**MSo**) production and a seasonal calving interval of 365 d. Animals were selected for the herd based on their overall Economic Breeding Index (**EBI**) values, particularly subindex values for milk and fertility, such that the overall EBI and herd subindex EBI for milk and fertility ranked in the top 5% and 10% nationally, respectively. Genetic evaluation data were taken from the annual EBI figures provided by the Irish Cattle Breeding Federation (https://www.icbf.com/). Animals selected were determined to have the genetic potential to enable the herd to produce a target of 625 kg MSo annually, a 50% increase from the national average in 2016 (Dillon et al., 2017). A mature cow liveweight of 620 to 630 kg was considered ideal to enable cows to have the intake capacity to consume sufficient energy to achieve a benchmark production efficiency of 1 kg MSo/kg liveweight. From 2016 to 2018, all primiparous replacement animals were selected, based on the aforementioned genetic criteria, from the existing herd in the UCD Lyons Farm. From 2019, all primiparous replacement animals selected were those been bred from cows within the study, to simulate a closed herd. Genetic selection of bulls focused on selecting sires with high milk subindex values and mature weights >620 kg. Preference was given to those with positive PTA values for chest and body depth, and functional traits, such as feet and mammary traits. Finally, bulls were ranked by fertility, prioritizing a negative calving interval PTA. This approach aimed to breed animals with the ability to meet both milk and reproductive performance targets imposed by the study. Whole-farm stocking rate and herd size from 2016 to 2024 are presented in Table 1. The available land base, herd size, and farm stocking rates varied throughout the study in response to the need to secure adequate grass silage quantities to meet herd requirements and to align with amendments in EU policies. Specifically, adjustments were required to accommodate the reclassification of organic N attributed to the herd (106 kg N/cow; DAFM, 2023a), in addition to a reduction in the maximum allowable organic N limits per hectare (220 kg N/ha; DAFM, 2023b). Replacement heifers are raised off farm on a separate land block and excluded from stocking rate calculations. After calving, the study herd was managed as one group. Cows were offered a basal diet of grazed perennial ryegrass (*Lolium perenne*; **PRG**), grass silage, and supplementary concentrate feed. The annual feed budget (**AFB**) in each year of the study is presented in Table 1. The targeted feed budget was based on the calculated herd energy requirements for 625 kg MSo (Nozière et al., 2018), the predicated grass growth potential of the farm and the estimated herd silage requirement for both the dry and lactating period. This subsequently influenced the required concentrate input, which was calculated as 1,500 kg (as-fed basis). Concentrate feed allowances were determined based on herd energy requirements and grazed grass availability, with the maximum daily rate offered being 8 kg (as fed) per cow. All cows were offered concentrate at an equal rate in each year of the study. Concentrate was offered at a flat rate of 8 kg/cow up to 60 DIM, and allowances thereafter were determined based on herd milk production and grazed grass and grass silage availability.

**Table 1:** Farm characteristics, including herd size and farm area, annual feed budget (kg DM/cow), and milk production performance (kg/cow), 2016–2024^1^

Item	Year									Mean
	2016	2017	2018	2019	2020	2021	2022	2023	2024	
Farm characteristic										
Herd size	58	60	59	58	57	57	57	56	57	57.69
Milking platform (ha)	17.58	17.65	17.65	17.52	17.43	17.43	17.43	17.43	17.43	17.5
Silage (ha)	7	7	7	7	7	7	7	7	10	7.3
Whole farm (ha)	24.58	24.65	24.65	24.52	24.43	24.43	24.43	24.43	27.43	24.84
Stocking rate (milking platform)	3.3	3.40	3.34	3.31	3.27	3.27	3.27	3.21	3.27	3.29
Stocking rate (whole farm)	2.36	2.43	2.39	2.37	2.33	2.33	2.33	2.29	2.08	2.32
Annual feed budget (kg DM/cow)										
Total	6,207	5,823	6,425	6,408	6,089	5,921	5,913	5,893	6,171	6,094
Grazed grass	3,031	3,043	2,758	3,410	3,356	3,221	2,567	2,736	2,260	2,931
Grass silage	1,855	1,512	2,301	1,691	1,873	1,443	2,004	1,876	2,601	1,906
Grass silage in lactation	1,278	982	1,815	1,149	1,281	980	1,455	1,246	1,907	1,343
Grass silage in dry period	577	530	486	542	592	463	549	630	694	563
Concentrate	1,321	1,267	1,366	1,308	1,237	1,258	1,342	1,282	1,310	1,299
Annual milk production										
Herd parity	2.6	2.8	2.9	2.9	3.2	3.1	3.2	3.5	3.6	3.1
Lactation length (d)	301	305	305	304	305	298	293	294	291	300
Milk yield (kg/cow)	7,407	7,466	6,790	7,381	7,503	7,733	7,010	7,108	6,748	7,238
Milk solids yield (kg/cow)	588	595	544	586	606	629	562	577	563	583
Milk protein (%)	3.56	3.66	3.62	3.60	3.59	3.64	3.62	3.56	3.60	3.60
Milk fat (%)	4.51	4.48	4.54	4.53	4.56	4.46	4.55	4.59	4.77	4.55

1 Annual feed budget includes grazed grass, grass silage, and concentrate allocated to lactating and dry cows within the year stated. Annual milk production figures include total milk yield (kg), milk solids yield (kg), and milk production efficiency metrics within the year stated. Milk solids per kg total DM = milk solids (kg) per cow ÷ total DM consumed per cow (kg). Milk solids per kg forage DM = milk solids (kg) per cow ÷ total forage DM consumed per cow (kg).

Cows were milked twice daily at 0700 h and 1500 h. Daily milk yield was determined from individual milk yield recorded during morning and evening milking using the Weighall milk meter system (Dairymaster, Causeway, Co. Kerry, Ireland). Milk composition was determined weekly from individual milk samples collected from consecutive evening and morning milkings. Samples were pooled in proportion to individual yield before being analyzed in a commercial milk laboratory (Independent Milk Laboratories, Cavan, Ireland). Concentrations of milk fat, protein, lactose, and SCC were determined using mid-infrared spectrophotometry (CombiFoss 5000, Foss Analytical A/S, Hillerød, Denmark). Milk solids yield (kg/d) was calculated as the sum of daily fat yield (kg/d) and protein yield (kg/d). Cow BW was recorded using an electronic scale (Dairymaster) after morning and evening milking, and a weekly mean was calculated for each cow. Body condition score was assessed every 2 to 3 wk by a single, trained operator using a scale of 1 to 5 with 0.25 increments, according to Edmonson et al. (1989), following morning milking. Breeding took place from the end of April to the beginning of July each year (10–12 wk). All breeding was done via artificial insemination. Initially, breeding was carried out once daily but increased to twice daily (each morning and evening after milking) from 2018. Cows were inseminated ~12 h after being observed in estrus, following the a.m./p.m. rule. Heat detection was carried out using a combination of visual aids (observation before milking each morning and evening, scratch cards) and activity sensor data.

The grazing area was managed as a rotational grazing system, whereby cows grazed full time as they calved from February onward. Approximately 10% of the milking platform was re-established annually with PRG and white clover (**WC**; *Trifolium repens*; from 2020 onward) such that paddocks were rejuvenated every 6 to 10 yr via minimum cultivation or direct drilling. Approximately 2 to 3 varieties of PRG were selected from the Irish Recommended List and the Pasture Profit Index (Tubritt et al., 2021) for seasonal growth and digestibility, along with medium leaf size WC. Twice weekly, individual pasture mass (n = 17 paddocks) was measured using the cut-and-weigh technique and the average farm cover recorded using the online grassland management application PastureBase Ireland (Hanrahan et al., 2017). An area (0.25 m^2^) was determined using a quadrat and cut using shears to 4 cm at 3 random locations in each paddock. Pregrazing pasture samples were collected, using a quadrat and shears, as new paddocks entered the grazing rotation. Samples were weighed and mixed, and a 150-g subsample collected for DM determination and subsequent nutritive analysis. Residency time within each paddock was dictated by a target postgrazing residual of 4 to 4.5 cm. Postgrazing residuals were measured daily using a rising plate meter (plate diameter 355 mm, area density 3.2 kg/m^2^; Jenquip, Feilding, New Zealand). Cows were allocated a target daily grazed grass allowance to achieve the target postgrazing residual and enable high levels of grass utilization (>85%). Herbage utilization (%) was calculated as [(pregrazing pasture mass − postgrazing pasture mass)/pregrazing pasture mass] × 100. Mean target grass allocations per cow for spring, summer, and autumn were 14.5, 14.1, and 9 kg DM respectively. Apparent grass intake per cow was calculated as [(pregrazing pasture mass − postgrazing pasture mass)/no. of cows grazing]/paddock residency time. Paddock residency time was greatest in the spring but reduced as the grazing season progressed. Mean paddock residency time from 2017 to 2024 was 1.9 d.

When required, grass silage was offered to all cows at an open feed face indoors. The herd were supplemented with grass silage when pasture and concentrate allocations were not sufficient to meet the herd energy requirements due to deficits in pasture availability. Pasture deficits occurred during periods of reduced pasture growth or unfavorable grazing conditions, resulting in herd pasture demand exceeding pasture growth (i.e., pasture supply). All cows were fed grass silage indoors at the end of grazing season and through the dry period. Cows were generally housed in early November, and most the herd was dried off by mid-December. Grass silage allowances in the dry period were determined based on silage quality and herd energy requirements (Nozière et al., 2018). During the far-off dry period, the dry cow diet consisted of grass silage and a dry cow mineral, trace element and vitamin premix. In the close-up dry period, cows were offered 2 kg/d of rolled barley and sources of chloride, including magnesium chloride and SoyChlor (Devenish Nutrition, Belfast, Northern Ireland) to ensure DCAD of 0 to +50 mEq/kg DM.

Inorganic nitrogen was applied monthly to paddocks from February to September in the form of urea or calcium ammonium nitrate at a maximum annual rate of 220 to 250 kg/ha (Table 2). Nitrogen application rates were adjusted annually in accordance with the best management practice guidelines outlined in Wall and Plunkett (2020) and sward clover content (WC % ≥20% = maximum 150 kg N/ha). Changes to the EU nitrates derogation legislation from 2024 reduced the maximum annual rate of N fertilizer from 250 to 220kg/ha in 2024 (DAFM, 2025; Table 1). Potassium, phosphorus, and lime applications were adjusted based on annual soil test results and pasture growth. In 2022 and 2023, P applications were restricted due to soil P levels exceeding 8.0 mg/L. Weather data were recorded by the Irish National Meteorological Service and were accessed from Casement Aerodrome (53°31′N, 6°44′W), a weather station ~6 km east of UCD Lyons Farm at similar elevation above sea level.

**Table 2:** Pasture DM yield on the milking platform (kg DM/ha), grazing characteristics, total nutrient applications (kg/ha), and feed (forage and concentrate) composition (g/kg DM), 2016–2024^1^

Item	Year									Mean
	2016	2017	2018	2019	2020	2021	2022	2023	2024	
Pasture DM yield (milking platform; kg DM/ha)										
Annual yield^2^	13,060	13,968	11,700	14,535	13,633	13,807	12,102	10,956	9,275	12,560
Silage harvested/ha	1,710	1,968	1,410	1,979	1,428	1,421	2,854	814	963	1,616
Grazed grass utilized/ha^3^	10,002	10,348	9,212	11,286	10,974	10,531	8,394	8,808	7,389	9,661
Grazing characteristic										
Pregrazing cover (kg DM/ha)	1,247	1,317	1,467	1,350	1,561	1,361	1,397	1,337	1,285	1,369
Utilization (%)	88.1	86.2	89.5	89.9	89.9	85.0	90.8	87.3	88.9	88.4
Days grazing (grass and silage)	279	279	245	275	278	287	297	272	253	274
Days grazing (grass only)	172	241	131	225	188	177	166	172	136	179
Nutrient application (kg/ha)										
Nitrogen, whole farm	219	237	229	231	195	188	159	155.7	177	199
Phosphorus (milking platform)	9.3	8.6	8.9	10	14.6	25.3	0	0	45	13.5
Potassium (milking platform)	31.7	44	112	120	84.7	95.3	29	47	112	75.1
Feed composition										
Grazed grass (g/kg DM)										
CP	—	236	196	232	193	169	167	173	167	191
NDF	—	426	397	414	405	397	446	433	446	420
Ether extract	—	31	32	31	31	32	28	27	28	30
OMD	—	866	871	873	867	849	847	839	847	857
Grass silage (g/kg DM)										
DM (%)	26.4	35.9	30.8	26.4	32.6	28.6	25.4	28.5	32.8	29.7
CP	123	146	156	157	124	141	156	143	121	140
NDF	443	405	462	484	474	469	460	496	489	465
Ether extract	33	32	42	49	41	28	29	28	32	35
Concentrate (g/kg)										
CP	161	150	149	149	148	148	157	140	140	149
NDF	276	223	191	191	225	225	220	244	244	227
Starch	263	290	283	283	285	285	287	272	272	280
UFL^4^	0.94	1.00	0.95	0.95	0.95	0.95	0.96	0.97	0.97	0.96

1 Grazing characteristics includes pregrazing cover (kg DM/ha), pasture utilization (%), and days grazing.

2 Annual herbage DM yield was recorded using the online pasture recording software PastureBase Ireland (Hanrahan et al., 2017).

3 Grazed grass utilized/ha = [total annual yield (kg DM/ha) − silage harvested (kg DM/ha)] × herbage utilization (%).

4 UFL = unit of energy for lactation.

Farm-gate nitrogen use efficiency (**NUE**) was calculated as the difference between N imports and N exports as described by Buckley et al. (2015) and Ryan et al. (2011). Farm N imports included chemical fertilizer, forage, concentrate feeds, calf milk replacer, and livestock. Imported forage included silage purchased in 2018 and 2024, and straw. Concentrate feeds included all concentrate fed to dairy cows and calf starter feed. Bull calves were assumed to have left the farm (sold) at 21 d. Heifer calves were assumed to have left the farm at 70 d and were reared from that point on at a separate farm until they returned ~6 wk before calving (contract rearing model). Atmospheric N deposition (kg N/ha) was assumed to be 9 kg N/ha (Hennessy et al., 2019). Biological N fixation (**BNF**; kg N/ha) by WC was estimated based on the following equation: N fixation (kg N/ha) = 8 × WC % − 77 (Enriquez-Hidalgo et al., 2016; Herron et al., 2021). Farm N exports comprised milk and livestock. Standard coefficients (ARC, 1994) were applied to milk production (kg) and livestock liveweight (kg) upon leaving the systems to calculate N exports in kilograms.

Grass, silage, and concentrate samples were analyzed from 2017 to 2024 in a commercial laboratory (FBA Laboratories, Waterford, Ireland) to determine the chemical composition of samples. Samples for analysis were pooled by week. Analytical methodology for determination of DM, CP, ash, NDF, ADF, water-soluble carbohydrates, and ether extract is described by Doran et al. (2022). Organic matter digestibility (**OMD**) of grass was determined by the CEL48-ND method as described by Kowalski et al. (2014). Briefly, a filter bag containing the sample (Ankom Technology, Macedon, NY) was incubated for 48 h at 39°C in a cellulase solution in the jars of a Daisy Incubator (Ankom Technology). After incubation, the bags with residues were extracted in a neutral detergent solution using an Ankom 220 Fiber Analyzer (Van Soest et al., 1991) and the OMD percentage calculated.

Continued efficiencies in grassland management are essential in higher-output systems, to ensure that sustainable intensification can be achieved to meet the food demands of a growing world population (Alexandratos and Bruinsma, 2012). Total annual grass growth averaged 12,560 kg DM/ha (Table 2), below the national target of 13,000 kg DM/ha (Teagasc, 2017) and the average of 13,500 kg DM/ha reported for Irish dairy farms using PastureBase Ireland (O'Donovan et al., 2022). Grassland performance in this study has been challenged by local site conditions. The soil on the farm is a clay loam, classified as a gray-brown podzolic soil (Lalor, 2004). Lyons Farm is a relatively dry site, with an average annual rainfall of 754 mm (SD = 81.3 mm) from 2016 to 2024, considerably lower than the national average of 1,288 mm from 1991 to 2020 (Curley et al., 2023). This lower rainfall, along with within-year variations in rainfall patterns, has important implications for herbage production. This was particularly evident in 2018 and 2024. Reduced annual rainfall and fluctuating rainfall patterns resulted in a high soil moisture deficit (**SMD**) in both years, with 42% of months recording SMD values <25 mm, the level at which grass growth begins to be negatively affected, resulting in seasonal periods of reduced growth (Met Éireann, 2020). In 2018, the summer drought was followed by record autumn growth rates, which enabled recovery in annual forage production (11,700 kg DM/ha). However, in 2024, consistently low grass growth rates in summer and autumn, due to the difficult growth conditions described, greatly reduced total herbage production in that year (9,275 kg DM/ha). Variation in pasture growth rates also creates challenges in utilizing available pasture. In 2022, periods of rapid compensatory growth in May and June led to pasture surpluses that exceeded the herd's intake capacity, resulting in increased silage harvested from the milking platform (Table 2). The reductions in total herbage production observed in this study have been partially offset by a high average herbage utilization rate of 88.4% (Table 2), above the national target of 75% (Teagasc, 2017).

In addition to interannual climatic variability, reduced N fertilizer use has negatively affected total herbage production and the quantity of grazed grass utilized per cow (Table 2). In this study, N fertilizer rates have reduced from 2019 to 2023. In 2020, WC establishment was prioritized on farm, subsequently resulting in lower fertilizer N applications (Wall and Plunkett, 2020). In 2024, changes in the EU nitrates derogation reduced the maximum N fertilizer rate on farm from 250 to 220 kg N/ha. Therefore, despite a reduced proportion of established WC in 2024, N applications were reduced relative to earlier years in the study. Reduced grazed grass utilized from 2022 to 2024 highlights the difficulty in balancing WC establishment and herbage production amid seasonal climate fluctuations. Variability in grazed grass availability (Table 1) and total grazing season length (Table 2) has led to fluctuations in feed supply and the amount of grass silage offered (1,443–2,601 kg DM/cow per year), challenging the ability of the system to meet the nutritional demands of higher-output grazing dairy cows. Total days spent grazing ranged from 245 in 2018, a year characterized by extreme weather and a national fodder crisis (Streethran et al., 2024), to 297 in 2022. Days grazing without grass silage supplementation ranged from 131 (2018) to 241 d (2017). Increasing the proportion of grazed grass in the diet is typically positively correlated with herd milk production, a trend generally supported by data from this study (Table 1). However, in 2022, cows spent the longest time grazing (297 d) and had relatively few days grazing with grass silage supplementation (166 d), but herd MSo production was below the 9-yr average. This may be due to reduced grass quality during that year, reflected in increased grass NDF and declining OMD compared with previous years. The decline in grass quality, potentially exacerbated by reduced N fertilizer applications, reduced the overall energy density of the diet, a trend that continued through 2023 and 2024. This highlights the sensitivity of dairy cow production within this system to forage quality (grass and grass silage), and the benefits of increasing days at grass are realized only if grass quality is sufficient to meet the metabolic demands of the grazing dairy cow.

Mean values of CP, NDF, and OMD concentrations for grazed grass in this study (Table 2) are similar to those reported by Murray et al. (2024) for grazing swards receiving 250 kg N/ha. Grass quality has generally been high, helping to support an average production of 583 kg MSo per cow within this system (Table 1). On average, 1,502 kg concentrate per cow (as fed) was used to support this level of MSo production, similar to the national average (1,358 kg/cow, as fed; Dillon et al., 2025). However, national MSo production is currently estimated to be 418 to 439 kg MSo per cow (Herron et al., 2022; Shallo et al., 2024), ~35% below the level of performance achieved in this study. As discussed, applications of inorganic N on the milking platform have decreased throughout this study.

This reduction in inorganic N may be reflected in the CP concentration of grass, which declined from an average of 236 g/kg DM in 2017 to 167 g/kg DM in 2024. The percentage of samples with less than 160 g/kg of DM CP has exceeded 30% for all years from 2021. A reduction in N intake and subsequently excretion by grazing dairy cows, through reduced grass CP concentrations, is likely to result in associated reductions in N losses, such as gaseous emissions and nitrate to inland water sources (de Vries et al., 2001; Mulligan et al., 2004; Cameron et al., 2013), providing an environmental benefit. From 2020, N fertilizer use was reduced to help establish and maintain WC in the grazing paddocks. This, combined with lower-CP concentrate feeds, decreased N inputs (from an average of 326 kg N from 2016 to 2020, to 262 kg N from 2021 to 2024) and led to a concurrent improvement in farm-gate NUE from 33% to 37.5% in the same period. Ryan et al. (2011) reported that as concentrate supplementation increased, so did N surplus per hectare. However, the average NUE from the 9 yr of this study was 35%, higher than figures reported for high-concentrate systems by Ryan et al. (2011; NUE 27.1%–30.8%). This highlights the potential for improved N utilization from higher-input dairy production systems through technical efficiency, reduced fertilizer use, and lower-CP concentrate feeds. However, some caution is required when reducing N fertilizer use, as estimates in this study indicate that BNF rates were relatively modest (4–19 kg N/ha). Reducing N fertilizer rates without sufficient deposition of N from legumes could negatively affect grass growth and milk production, reducing farm-gate N exports and NUE.

Genetic selection has an important role to play in a higher-input, higher-output spring-calving dairy production system. Animals in this study require the genetic potential to efficiently consume DM and convert it to MSo, while maintaining body condition. A mature cow liveweight of 620 to 630 kg BW is considered ideal, as heavier cows (610 kg BW) are able to produce more milk and MSo than lighter cows (550–590 kg BW); however, this production advantage diminishes when cow BW exceeds 650 kg (Berry and Evans, 2022). Previous work by our research group showed that cows selected for higher milk PTA were more efficient at producing MSo than cows with lower milk PTA (Brady et al., 2021; Doran et al., 2022). Therefore, bulls selected for this study were selected for positive milk, fat, and protein yields. Production traits of bulls selected were ensured to be greater than the average PTA of the heifers and cows chosen to breed replacements that year. This strategy provides a genotype with the ability to increase milk yield while maintaining milk composition, as under the Irish milk pricing system farmers are paid for protein and fat yield and penalized for milk yield. In this study, the challenge is to provide genetic support to attain a milk production target of 625 kg MSo, while maintaining herd fertility. This emphasis on fertility is reflected in the phenotypic performance of the herd. Average submission rate at 21 d (91.5%), first service conception rate (63.5%), and 6-wk pregnancy rate (76.4%) over 9 yr have all exceeded national fertility targets. To achieve high milk production output and a calving interval of 365 d, selection emphasis must be placed on the traits and subindices of the EBI considered most important to the system rather than the overall EBI value of the bull or cows. Despite the negative weightings on milk production traits within the EBI, the balanced selection for both milk and fertility within the bull team has led to the overall herd EBI ranking within the top 1% of all herds nationally from 2016 to 2022.

A primary financial objective of higher-output grazing systems is to achieve profitability that matches or exceeds those of a benchmark highly efficient low-concentrate, spring-calving system, producing 465 kg MSo/cow with 500 kg purchased feed per cow, at a whole-farm stocking rate of 2.39 livestock units per hectare (Teagasc, 2020). Figure 1 shows differences in net margin per hectare between this study, using the average 9-yr performance data from this study, and this benchmark low-concentrate system, according to a range of milk and feed prices. We note that both systems generate broadly similar whole-farm levels of profitability despite the UCD system having 15% fewer cows on the same land area. As expected, the higher-output system employed in this study was more sensitive to fluctuations in milk and feed prices. Under low milk price or high feed cost conditions, this study tended to underperform the benchmark system, whereas in favorable market environments it showed potential for superior profitability. Between 2013 and 2024 in Ireland, the annual base milk price has averaged above 35 cents/L in 7 of the 12 yr, and concentrate price has exceeded €400/t in only 2 years (CSO, 2025). Consequently, the upside risk has generally favored the higher-output system in recent years, which may have contributed to the national trend toward increased concentrate supplementation (Dillon et al., 2025).

**Figure 1:**
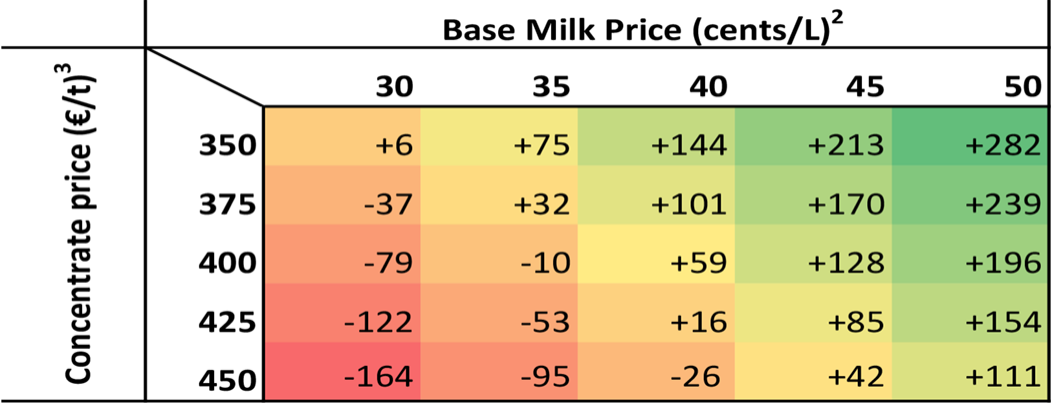
Difference in net margin per hectare of the system examined relative to benchmark low-concentrate system according to milk and concentrate price, with all other prices and costs held constant at 2024 levels. Benchmark was spring-calving pasture-based system averaging 465 kg MSo/cow with 500 kg concentrates/cow and a whole-farm stocking rate of 2.39 cows/ha (Teagasc, 2020). Milk price shown at a base milk composition of 3.3% protein and 3.7% butterfat and assuming SCC <200,000, protein-to-butterfat price ratio of 2:1, and volume-related deduction of €0.04/L. Concentrate was 16% CP dairy blend.

Results from this study show that a higher-output grass-based spring milk production system can be financially competitive when built on a foundation of appropriate genetics, good grassland management, and meeting performance targets. Grassland performance has been challenged by annual variations in weather and rainfall patterns, and reduced N fertilizer application rates. Despite this, high levels of grazed grass utilization were achieved, and grass quality was not negatively affected. Milk production per cow was more than ~30% higher than the national average, and herd reproductive performance over the 9 yr of the study exceeded national fertility targets for spring-calving systems. Future research should focus on strategies to improve interyear performance consistency, particularly in responding to weather-related variation in grass quality and quantity. This may include more flexible and responsive concentrate supplementation approaches to offset yield depressions during periods of constrained herbage availability.
